# Evaluating the efficacy and safety of macrolide therapy in primary ciliary dyskinesia: A systematic review

**DOI:** 10.1097/MD.0000000000050263

**Published:** 2026-08-21

**Authors:** Masoumeh Ghasempour Alamdari, Paniz Pourpashang, Simin Khayatzadeh Kakhki, Malihe Sadat Rahimi Azghadi, Niloufar Ghanbari, Parisa Ashournia, Arezoo Akhlaghi, Ghazal Shariatpanahi

**Affiliations:** a Department of Pediatric Pulmonology, Bahrami Hospital, School of Medicine, Tehran University of Medical Sciences, Tehran, Iran; b Department of Pediatric Nephrology, Bahrami Hospital, School of Medicine, Tehran University of Medical Sciences, Tehran, Iran; c Division of Pediatric Neurology, Bahrami Children Hospital, School of Medicine, Tehran University of Medical Sciences, Tehran, Iran; d Department of Pediatric Endocrinology, Bahrami Hospital, School of Medicine, Tehran University of Medical Sciences, Tehran, Iran; e Emergency Division, Bahrami Hospital, School of Medicine, Tehran University of Medical Sciences, Tehran, Iran; f Department of Pediatric Allergy and Clinical Immunology, Bahrami Hospital, School of Medicine, Tehran University of Medical Sciences, Tehran, Iran; g Department of Pediatric Infectious Diseases, Bahrami Hospital, School of Medicine, Tehran University of Medical Sciences, Tehran, Iran.

**Keywords:** anti-inflammatory therapy, azithromycin, macrolides, primary ciliary dyskinesia, respiratory exacerbations, systematic review

## Abstract

**Background:**

Primary ciliary dyskinesia (PCD) is a rare genetic disorder characterized by impaired ciliary function, defective mucociliary clearance, recurrent respiratory infections, and progressive respiratory morbidity. Macrolides, particularly azithromycin, have antibacterial, anti-inflammatory, and immunomodulatory properties and may therefore provide therapeutic benefit in PCD. This systematic review evaluated the efficacy and safety of macrolide therapy in patients with PCD.

**Methods:**

This systematic review was conducted in accordance with the Preferred Reporting Items for Systematic Reviews and Meta-Analyses 2020 guidelines and was prospectively registered in International Prospective Register of Systematic Reviews (CRD42024553793). PubMed, Scopus, Web of Science, Embase, Cochrane, and Google Scholar were searched for studies published from January 1, 1990, to June 18, 2024. Eligibility criteria were defined using the population, intervention, comparator, outcomes, and study design framework. Study selection, data extraction, and risk of bias assessment were performed independently by 3 reviewers. A meta-analysis was not performed because of substantial methodological heterogeneity across the included studies.

**Results:**

Of 765 records identified, 9 studies met the inclusion criteria. These included 2 in vitro studies and 7 clinical studies, comprising case reports, case series, retrospective observational studies, and 1 randomized, placebo-controlled phase 3 trial. Across the clinical studies, sample sizes ranged from 1 to 90 participants. Overall, the available evidence suggested that macrolide therapy, particularly azithromycin, may reduce respiratory exacerbations and improve selected clinical symptoms in patients with PCD. Mechanistic in vitro evidence further indicated that azithromycin may reduce pro-inflammatory cytokine production and promote epithelial barrier repair. However, the included studies varied considerably in design, population characteristics, treatment protocols, and outcome definitions.

**Conclusion:**

Macrolides, especially azithromycin, may have a beneficial role in the management of PCD by reducing respiratory exacerbations, improving selected symptoms, and modulating airway inflammation. Nevertheless, the certainty of evidence remains limited by methodological heterogeneity, small sample sizes, and the scarcity of randomized controlled trials. Further large-scale, well-designed, PCD-specific clinical studies are required to confirm these preliminary findings and establish standardized treatment protocols.

## 1. Introduction

Primary ciliary dyskinesia (PCD) is a rare, genetically heterogeneous disorder characterized by abnormal ciliary structure or function, resulting in impaired mucociliary clearance. This defect predisposes affected individuals to recurrent respiratory infections, chronic otitis media, bronchiectasis, and, in approximately half of cases, situs inversus.^[1,2]^ Because effective mucociliary clearance is essential for removing inhaled pathogens and debris from the airway surface, ciliary dysfunction contributes to persistent airway inflammation and progressive respiratory morbidity.^[3]^ Patients with PCD commonly experience chronic wet cough, recurrent lower respiratory tract infections, bronchiectasis, and reduced lung function, creating substantial clinical challenges in long-term disease management.^[4,5]^

Macrolides are antibiotics with well-recognized antibacterial, anti-inflammatory, and immunomodulatory properties. In addition to their antimicrobial effects, macrolides can modulate immune responses, reduce the production of pro-inflammatory cytokines, and influence airway epithelial function.^[6,7]^ Long-term macrolide therapy has been associated with fewer respiratory exacerbations and improved clinical outcomes in several chronic airway diseases, including cystic fibrosis, non-cystic fibrosis bronchiectasis, and chronic obstructive pulmonary disease.^[8–13]^ However, extrapolating these findings to PCD requires caution. Unlike cystic fibrosis, which is primarily related to ion transport dysfunction, and chronic obstructive pulmonary disease, which is largely driven by acquired airway injury, PCD results from primary defects in ciliary motility or structure. These disease-specific mechanisms may influence both airway inflammation and response to macrolide therapy.^[14]^

The clinical management of PCD remains complex because of its broad genetic diversity and variable clinical phenotype.^[15]^ Different disease-causing mutations and ciliary ultrastructural defects may lead to varying degrees of mucociliary impairment, airway inflammation, epithelial injury, and microbial colonization. This heterogeneity may partly explain why treatment responses, including responses to macrolides, differ among patient subgroups. Current management is mainly supportive and focuses on airway clearance, treatment of acute infections, monitoring of lung function, and prevention of disease progression.^[15]^ However, the chronic inflammatory burden and recurrent infectious complications associated with PCD highlight the need for therapies that may modify the disease course. In this context, macrolides, particularly azithromycin, have received increasing attention as potential long-term therapeutic agents in PCD.^[16,17]^

Despite emerging evidence, the role of macrolide therapy in PCD remains insufficiently defined. There is no clear consensus regarding optimal patient selection, dosing regimen, treatment duration, or long-term safety monitoring. The available evidence is also limited by heterogeneity in study design and includes in vitro experiments, case reports, case series, retrospective studies, and a small number of controlled clinical investigations. Therefore, a comprehensive synthesis of the existing literature is needed to clarify the potential benefits and limitations of macrolide therapy in this population. This systematic review aimed to evaluate the efficacy and safety of macrolide therapy, particularly azithromycin, in patients with PCD and to summarize the clinical and mechanistic evidence relevant to future research and practice.

## 2. Materials and methods

### 2.1. Protocol and reporting

This systematic review was conducted in accordance with the Preferred Reporting Items for Systematic Reviews and Meta-Analyses 2020 statement.^[18]^ The review protocol was prospectively registered in the International Prospective Register of Systematic Reviews (registration number: CRD42024553793). The methods were designed to ensure transparency, reproducibility, and comprehensive identification of the available evidence on macrolide therapy in PCD. Because this study synthesized previously published data and involved no direct interaction with human participants or access to identifiable individual-level data, ethics committee or institutional review board approval and informed consent were not required.

### 2.2. Eligibility criteria

Eligibility criteria were defined using the population, intervention, comparator, outcomes, and study design (PICOS) framework,^[19]^ which specifies the PICOS of interest. This framework was used to guide study selection and to ensure that included studies were directly relevant to the review question.

### 2.3. Population

Studies involving patients with PCD or human-derived PCD samples were eligible, regardless of age or sex. Animal studies and studies based on nonhuman samples were excluded. In vitro studies using human-derived PCD cells or samples were retained when they provided mechanistic evidence relevant to macrolide therapy.

### 2.4. Intervention

Eligible studies evaluated the direct administration or experimental exposure of macrolides, including azithromycin, clarithromycin, erythromycin, or other macrolide agents. No restrictions were applied regarding dose, route of administration, treatment duration, or treatment schedule. Studies in which macrolides were administered as part of a combined intervention were excluded when the independent effect of the macrolide could not be distinguished from that of other therapies.

### 2.5. Comparator

Studies were eligible regardless of the comparator used. Comparators included placebo, standard care, active control interventions, untreated controls, or within-subject before-and-after comparisons. For in vitro studies, untreated or vehicle-treated cells were considered acceptable comparators.

### 2.6. Outcomes

The primary outcomes were clinical indicators of macrolide efficacy in PCD, including respiratory exacerbations, respiratory symptoms, lung function parameters, and other disease-related clinical outcomes. Secondary outcomes included safety and tolerability, adverse events, inflammatory biomarkers, epithelial barrier-related outcomes, ciliary function, and other mechanistic or laboratory markers relevant to macrolide activity.

### 2.7. Study design

We considered randomized controlled trials, observational studies, case-control studies, cross-sectional studies, case reports, and case series. In vitro studies were also included when they used human-derived PCD samples and provided mechanistic evidence related to macrolide effects on inflammatory pathways, epithelial repair, or ciliary function. Letters, editorials, commentaries, opinion articles, narrative reviews, systematic reviews, animal studies, conference abstracts, and studies without original relevant data were excluded.

### 2.8. Information sources

A comprehensive literature search was conducted in PubMed, Scopus, Web of Science, Embase, Cochrane, and Google Scholar. The search covered studies published from January 1, 1990, to June 18, 2024. No language restrictions were applied. Non-English studies were assessed using English abstracts or translations when required. Gray-literature sources, including ClinicalTrials.gov, the World Health Organization International Clinical Trials Registry Platform, allconferences.com, and OpenGrey, were also searched. In addition, the reference lists of relevant articles were manually screened to identify potentially eligible studies not captured through the electronic search.

### 2.9. Search strategy

The search strategy was tailored to each database using a combination of controlled vocabulary terms and free-text keywords related to PCD and macrolides. Medical Subject Headings terms were used for PubMed, and the Embase life science thesaurus (Emtree) was used for Embase where applicable. Additional keywords and abbreviations were identified through consultation with subject-matter experts to improve the sensitivity of the search. Full search strategies for all databases are provided in Tables S1–S6, Supplemental Digital Content 1.

### 2.10. Selection process

All retrieved records were imported into EndNote 17, and duplicate records were removed before screening. Two reviewers independently screened titles and abstracts against the predefined eligibility criteria. Full texts of potentially eligible studies were then assessed independently by 2 reviewers. Any disagreements were resolved through discussion, and unresolved discrepancies were adjudicated by the corresponding author.

### 2.11. Data collection process

Data extraction was performed independently by 3 reviewers, namely, GSH, MGA, and PP, using a standardized Excel-based extraction form. Extracted data were checked for accuracy and consistency across reviewers. When essential data were unclear or missing, the review protocol allowed for contact with corresponding authors using a 3-email approach. However, no corresponding authors ultimately needed to be contacted because all required data were available in the published reports. Therefore, the number of author responses received was 0.

### 2.12. Data items

The extracted data included author name, year of publication, country, study design, sample size, participant or sample characteristics, type of macrolide, dose, and treatment regimen where applicable, comparator, follow-up duration, clinical outcomes, safety outcomes, mechanistic findings, and the main conclusions reported by each study.

### 2.13. Risk of bias assessment

Risk of bias was assessed according to study design. The Cochrane Risk of Bias 2 tool was used for randomized controlled trials. The Newcastle–Ottawa scale was used for observational studies, including cohort and case-control designs, where applicable. For case reports, case series, and in vitro studies, methodological limitations were considered narratively because these designs are not fully captured by standard randomized or observational risk of bias tools.

### 2.14. Data synthesis

A quantitative meta-analysis was planned if the included studies were sufficiently homogeneous in terms of study design, population, intervention, comparator, and outcome definitions. However, because of substantial clinical and methodological heterogeneity across the included studies, a meta-analysis was not performed. Findings were therefore synthesized narratively, with studies grouped according to design and evidence type, including in vitro studies, case reports or case series, retrospective studies, and randomized controlled evidence.

## 3. Results

### 3.1. Study selection

#### 3.1.1. Study selection process

**Figure 1.**
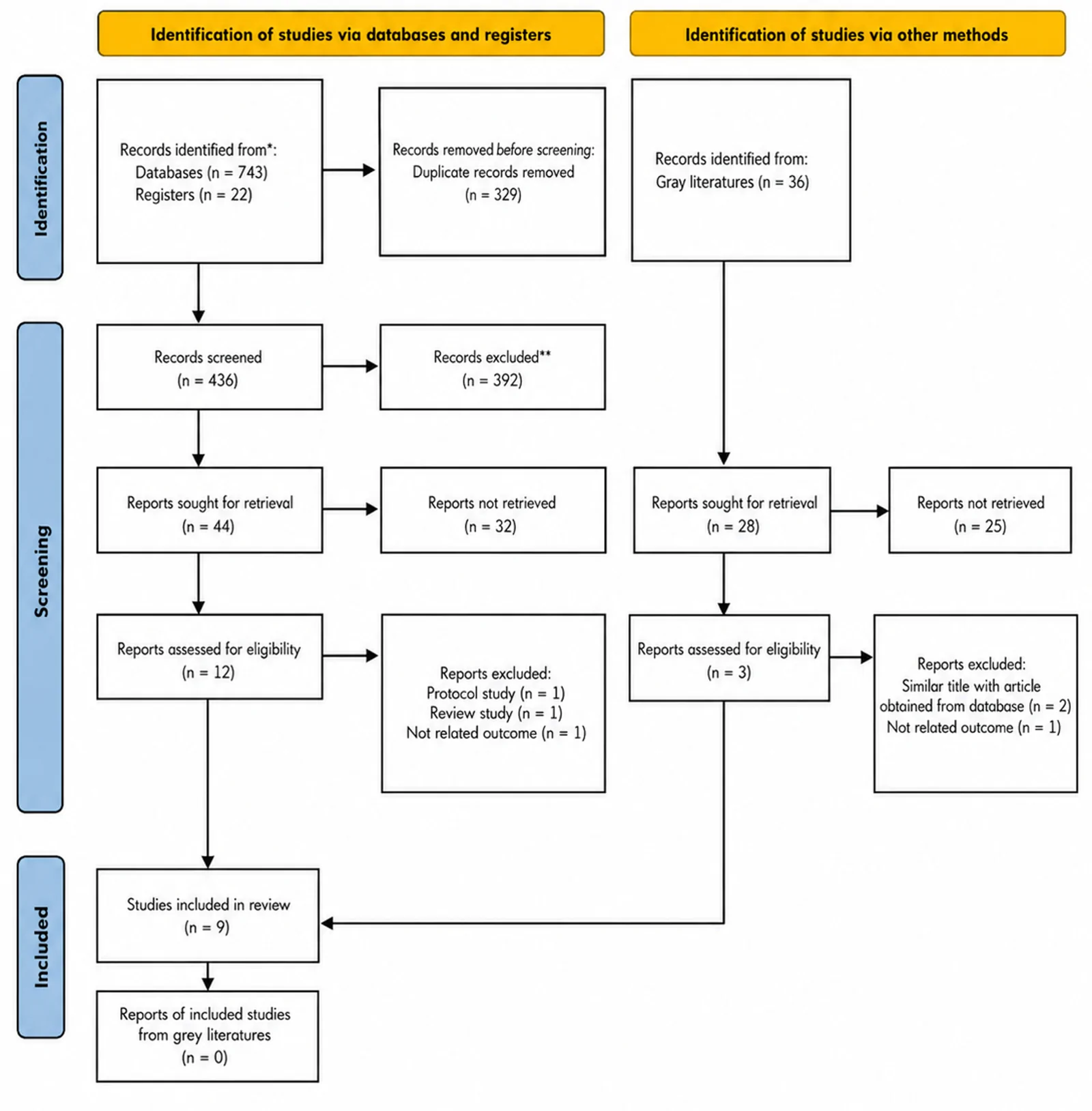
PRISMA 2020 flow diagram of the study selection process. n = number of records, PRISMA = Preferred Reporting Items for Systematic Reviews and Meta-Analyses.

A detailed overview of the study selection process is presented in Figure 1. The systematic searches identified a total of 765 records. PubMed yielded 192 records, Scopus yielded 246, Web of Science yielded 150, Embase yielded 100, the Cochrane Library yielded 35, and Google Scholar yielded 20. An additional 22 records were identified through gray-literature sources, including ClinicalTrials.gov, the World Health Organization International Clinical Trials Registry Platform, AllConferences, and OpenGrey. After the removal of 329 duplicate records, the titles and abstracts of the remaining 436 records were independently screened by 2 reviewers. Of these, 427 records were excluded because they did not meet the predefined eligibility criteria. Consequently, 9 studies were included in the qualitative synthesis.^[16,17,20–26]^

#### 3.1.2. Study characteristics

The main characteristics of the included studies are presented in Table 1. The 9 included studies were conducted in China, Japan, France, the Netherlands, Switzerland, and the Czech Republic. Two studies were in vitro investigations using human-derived PCD samples, whereas the remaining studies included case reports, case series, retrospective observational studies, and 1 multicenter, double-blind, randomized, placebo-controlled phase 3 trial. Across the clinical studies, sample sizes ranged from 1 to 90 participants. The evaluated macrolides included azithromycin, clarithromycin, erythromycin, or combinations of macrolide agents. The outcomes reported across studies included respiratory exacerbations, clinical symptoms, radiological findings, lung function, inflammatory markers, epithelial repair, ciliary function, and safety outcomes.

**Table 1:** Description of included studies characteristics.

Author’s name (year)	Country	Study aim/ Design	Sample size	Macrolides type	Major Results
Varenyiova et al (2023)	Czech Republic	AZT effects on cytokine levels in nasal epithelial cells in PCD/in vitro study	3	AZT	AZT administration demonstrated the ability to mitigate inflammation in both BC and MCE cells, while also promoting cell proliferation specifically in PCD BCs.
Zhang et al (2022)	China	CCNO mutation in a case with PCD/case report	1	AZT	A follow-up chest CT scan performed after 2 months of oral AZT therapy revealed a noticeable reduction in the size of the lung lesion in the lower left lobe.
Mori et al (2022)	Japan	absence of SPEF2 protein expression/case report	1	AZT	macrolide therapy reduced respiratory symptoms
Guan et al (2022)	China	Assess AZT treatment effects on disease progression in pediatric PCD patients/retrospective follow-up study	71	AZT	This treatment strategy has the potential to decrease the frequency of respiratory infections while also potentially stabilizing the progression of pulmonary diseases.
Dabrowski et al (2021)	Switzerland	Effects of non-aminoglycoside compounds, including AZT, on PTC readthrough in PCD/in vitro study	4	AZT	Significant effects of AZT on translational readthrough of premature termination codons (PTC readthrough)
Kobbernagel et al (2020)	Netherlands	Efficacy and safety of AZT in patients with PCD/multicenter, double-blind, randomized, placebo-controlled phase 3 trial	90	AZT	AZT maintenance therapy significantly reduced respiratory exacerbations compared with placebo (rate ratio, 0.45; 95% CI, 0.26–0.78; *P* = .004), corresponding to approximately 55% fewer exacerbations over 6 months. Serious adverse events were infrequent and comparable between groups, although loose stools or diarrhea were more common with AZT than with placebo (23% vs. 5%).
Massiot et al (2018)	France	Effect of long-term AZT on the annual number of pulmonary exacerbations in children with PCD/retrospective study	31	AZT	clinical improvement of PCD children treated by long-term AZT
Kido et al (2016)	Japan	Evaluation of macrolides administration in 2 patients with PCD/case series	2	Clarithromycin, Erythromycin, and AZT	Two differences in response to these drugs were observed in 2 cases.
Tsubouchi et al (2015)	Japan	Effects of clarithromycin treatment in a PCD case/case report	1	Clarithromycin	clarithromycin may be considered one of the agents for treating PCD

AZT = azithromycin, BC = bronchial club, CCNO = Cyclin O, MCE = mucin-producing ciliated epithelial cells, PCD = primary ciliary dyskinesia, PTC = premature termination codon, SPEF2 = sperm flagellar and cilia associated 2.

#### 3.1.3. Results of in vitro studies

Varenyiova et al evaluated the effects of azithromycin on proliferation and inflammatory pathways in nasal epithelial cells isolated from patients with PCD. The study showed that PCD respiratory epithelium exhibited increased inflammatory activity and impaired proliferative capacity. Azithromycin reduced pro-inflammatory cytokine production and promoted epithelial repair, suggesting a potential role in modulating airway inflammation and supporting epithelial barrier renewal in PCD.^[20]^

Dabrowski et al investigated non-aminoglycoside compounds for their ability to induce translational readthrough of premature termination codons in PCD-related genes. The study compared these compounds with aminoglycosides and assessed their effects on readthrough efficiency, cell viability, and ciliary function. Although non-aminoglycoside compounds showed lower readthrough efficiency than aminoglycosides, they were less harmful to cell viability. Azithromycin was identified as one of the promising non-aminoglycoside candidates. These findings suggest that macrolide-related compounds may have potential mechanistic relevance in selected genetic contexts, although their clinical applicability remains uncertain and requires further validation.^[16]^

#### 3.1.4. Results of case reports and case series

Four case reports and case series, mainly from China and Japan, evaluated macrolide therapy in a total of 5 patients with PCD, including both adolescent and adult patients.^[21,24–26]^ The treatments administered included azithromycin, clarithromycin, erythromycin, or combinations of macrolides. Overall, most reports described improvement in respiratory symptoms, recurrent respiratory infections, cough, or radiological findings after macrolide therapy. However, the magnitude and consistency of response varied between patients. One report described an unsuccessful response to combined clarithromycin, erythromycin, and azithromycin therapy, suggesting that treatment response may differ according to patient-specific factors, including age, clinical phenotype, genetic background, or underlying ciliary defects.

#### 3.1.5. Results of studies with larger sample sizes

Three studies with comparatively larger sample sizes evaluated the clinical effects of azithromycin in patients with PCD.^[17,22,23]^ Massiot et al conducted a retrospective study of 31 children with PCD, with a mean age of 11 years, to assess the effect of long-term azithromycin treatment on pulmonary exacerbations. After 1 year of treatment, the annual frequency of pulmonary exacerbations decreased significantly, from 1.7 ± 2.7 exacerbations per year before treatment to 0.5 ± 1.0 exacerbations per year after treatment. Ear, nose, and throat symptoms also improved. However, no measurable change was observed in the rate of lung function decline, airway colonization, or emergence of antibiotic resistance during the treatment period.^[23]^

In a retrospective follow-up study of pediatric patients with PCD, Guan et al compared clinical outcomes between patients receiving azithromycin and those receiving control treatment. Patients treated with azithromycin had a significantly lower frequency of respiratory exacerbations than controls, with 1.4 ± 0.8 versus 3.0 ± 2.1 exacerbations per year, respectively (*P* = .001). A lower proportion of patients in the azithromycin group also experienced exercise intolerance. Although forced expiratory volume in 1 second and forced vital capacity percent-predicted values were higher in the azithromycin group than in the control group, these differences were not statistically significant.^[22]^

Kobbernagel et al conducted a multicenter, double-blind, randomized, placebo-controlled phase 3 trial evaluating azithromycin maintenance therapy in patients with PCD. The trial showed that azithromycin significantly reduced respiratory exacerbations compared with placebo and was generally well tolerated. Gastrointestinal symptoms, particularly loose stools or diarrhea, were reported more frequently in the azithromycin group. Detailed effect estimates and adverse-event data are provided in Table 1.^[17]^

## 4. Discussion

This systematic review synthesized the available evidence on the efficacy and safety of macrolide therapy in patients with PCD. The included evidence comprised in vitro studies, case reports, case series, retrospective observational studies, and 1 randomized, placebo-controlled phase 3 trial. Overall, the findings suggest that macrolides, particularly azithromycin, may reduce respiratory exacerbations and improve selected clinical symptoms in patients with PCD. However, these findings should be interpreted cautiously because the evidence base remains limited by substantial heterogeneity in study design, patient characteristics, treatment regimens, outcome definitions, and follow-up duration.

The potential benefit of macrolide therapy in PCD is biologically plausible. Previous studies in chronic airway diseases, including cystic fibrosis, non-cystic fibrosis bronchiectasis, and chronic obstructive pulmonary disease, have reported that long-term azithromycin therapy can reduce respiratory exacerbations and improve selected clinical outcomes.^[27–32]^ Nevertheless, direct extrapolation from these conditions to PCD should be avoided. PCD is caused by primary abnormalities in ciliary structure or function, whereas cystic fibrosis is primarily related to ion transport dysfunction, and non-cystic fibrosis bronchiectasis is often a consequence of acquired or secondary airway injury. These pathophysiological differences may influence airway inflammation, microbial colonization, mucociliary clearance, and treatment response.

In PCD, impaired ciliary motility leads to defective mucociliary clearance and retention of airway secretions. This promotes recurrent respiratory infections and chronic airway inflammation, which may progressively damage the respiratory epithelium. Common bacterial pathogens reported in PCD include *Haemophilus influenzae*, *Streptococcus pneumoniae*, *Moraxella catarrhalis*, *Staphylococcus aureus*, and *Pseudomonas aeruginosa*.^[33]^ Although the inflammatory profile of PCD is not fully characterized, increased interleukin-8/CXCL8 production has been reported in some studies.^[34]^ In this context, the anti-inflammatory and immunomodulatory effects of macrolides may be clinically relevant.

The in vitro evidence included in this review provides mechanistic support for a possible role of azithromycin in PCD. Varenyiova et al reported that azithromycin reduced pro-inflammatory cytokine production and promoted epithelial barrier repair in nasal epithelial cells derived from patients with PCD.^[20]^ These findings are consistent with broader experimental evidence suggesting that azithromycin may help preserve airway epithelial integrity by influencing tight junction regulation and supporting epithelial repair processes.^[35,36]^ However, these mechanistic findings should be viewed as hypothesis-generating rather than definitive evidence of clinical efficacy.

Macrolides may exert beneficial effects through several complementary mechanisms. Their anti-inflammatory activity may involve downregulation of pro-inflammatory mediators, including interleukin-8 and tumor necrosis factor-alpha, both of which are relevant to chronic airway inflammation.^[20,37]^ Macrolides may also influence immune cell function by reducing neutrophil recruitment and modulating macrophage activity, thereby contributing to the resolution of persistent airway inflammation.^[14,16,20,24,29]^ In addition, some studies suggest that macrolides may affect epithelial repair and mucociliary function. However, in PCD, any potential effect on mucociliary clearance must be interpreted carefully because the primary defect lies in ciliary structure or motility. Therefore, although macrolides may improve the inflammatory airway environment, their ability to directly correct ciliary dysfunction remains uncertain.^[22]^

Several mechanisms proposed in the broader macrolide literature, including epigenetic modulation and histone methylation, remain speculative in the context of PCD. These mechanisms have been discussed in respiratory disease models but were not directly evaluated in the PCD-specific clinical evidence included in this review.^[4,38,39]^ Accordingly, they should not be considered established mechanisms of benefit in PCD. Future mechanistic studies using human-derived PCD airway models are needed to clarify whether these pathways are relevant to macrolide response in this disease.^[40,41]^

The strongest clinical evidence came from the randomized, placebo-controlled phase 3 trial by Kobbernagel et al, which showed that azithromycin maintenance therapy reduced respiratory exacerbations compared with placebo and was generally well tolerated.^[17]^ However, the available randomized evidence remains limited to a single trial; therefore, additional multicenter studies with longer follow-up are needed to confirm the durability of benefit, effects on lung function, and long-term safety.

The retrospective studies also suggested potential clinical benefit. Massiot et al reported a significant reduction in annual pulmonary exacerbations after 1 year of long-term azithromycin therapy in children with PCD, along with improvement in ear, nose, and throat symptoms.^[23]^ Guan et al similarly reported fewer respiratory exacerbations among pediatric patients receiving azithromycin than among controls.^[22]^ However, both studies were observational and therefore susceptible to confounding, selection bias, and differences in baseline disease severity. These limitations reduce the certainty with which causal conclusions can be drawn.

The evidence from case reports and case series was generally supportive but highly limited. Most reports described improvement in respiratory symptoms, recurrent infections, cough, or radiological findings after macrolide therapy. However, 1 report described an unsuccessful response to combined macrolide therapy, highlighting potential variability in treatment response. This variability may reflect differences in age, disease severity, genotype, ciliary ultrastructural defects, microbial colonization, or inflammatory phenotype. Because PCD is genetically heterogeneous, future studies should consider genotype- or phenotype-stratified analyses to determine whether specific patient subgroups are more likely to benefit from macrolide therapy.

Long-term safety remains a major consideration, particularly in pediatric patients. Although Massiot et al did not observe a significant emergence of antibiotic resistance during the treatment period,^[23]^ prolonged macrolide exposure may increase the risk of resistant bacterial strains and could complicate future treatment options. In the randomized trial by Kobbernagel et al, serious adverse events were infrequent and comparable between groups, although loose stools or diarrhea occurred more frequently among patients receiving azithromycin.^[17]^ These findings indicate that azithromycin may be generally well tolerated, but longer follow-up is required to evaluate antimicrobial resistance, gastrointestinal adverse effects, microbiome changes, and other potential long-term safety concerns.

To our knowledge, this is the first systematic review to synthesize clinical and mechanistic evidence on macrolide therapy in PCD. The main strengths of this review include a comprehensive search strategy, inclusion of multiple electronic databases and gray-literature sources, prospective International Prospective Register of Systematic Reviews registration, use of the Preferred Reporting Items for Systematic Reviews and Meta-Analyses 2020 framework, and structured eligibility criteria based on the PICOS approach. These methodological features enhance the transparency and reproducibility of the review.

This review also has several limitations. First, substantial clinical and methodological heterogeneity precluded meta-analysis. The included studies differed in design, sample size, population characteristics, type and duration of macrolide therapy, comparator groups, and outcome definitions. Second, the evidence base was small, and most clinical studies had limited sample sizes. Case reports and case series, although useful for generating hypotheses, are inherently prone to bias and have limited generalizability. Third, only one randomized controlled trial was available, which restricts the strength of the overall evidence. Fourth, the inclusion of in vitro studies provided useful mechanistic insights but limited direct clinical applicability. Fifth, the included studies were geographically concentrated in Asia and Europe, which may limit generalizability to other regions with different genetic backgrounds, healthcare systems, microbial exposures, and treatment practices. Finally, because a meta-analysis was not feasible, formal assessment of publication bias could not be performed.

Overall, the available evidence suggests that macrolides, especially azithromycin, may have a beneficial role in reducing respiratory exacerbations and improving selected clinical outcomes in PCD. However, the current evidence is not sufficient to establish standardized treatment recommendations. Further large-scale, multicenter, PCD-specific randomized trials are needed to confirm efficacy, define optimal patient selection, determine appropriate dosing and treatment duration, and assess long-term safety. Future studies should also incorporate standardized outcome measures, genotype- or phenotype-based subgroup analyses, and systematic monitoring of antimicrobial resistance.

## 5. Conclusion

Preliminary evidence suggests that macrolides, particularly azithromycin, may reduce respiratory exacerbations and improve selected clinical symptoms in patients with PCD. Mechanistic studies also suggest potential anti-inflammatory and epithelial barrier-related effects. However, the certainty of the evidence remains limited by small sample sizes, methodological heterogeneity, limited randomized evidence, and the absence of standardized treatment protocols. Well-designed, multicenter, PCD-specific randomized trials with longer follow-up are needed to establish the efficacy, safety, optimal use, and long-term consequences of macrolide therapy in this population.

## Author contributions

**Conceptualization:** Ghazal Shariatpanahi.

**Data curation:** Simin Khayatzadeh Kakhki, Ghazal Shariatpanahi.

**Formal analysis:** Simin Khayatzadeh Kakhki.

**Investigation:** Masoumeh Ghasempour Alamdari, Paniz Pourpashang, Malihe Sadat Rahimi Azghadi, Niloufar Ghanbari, Parisa Ashournia, Arezoo Akhlaghi.

**Methodology:** Niloufar Ghanbari, Parisa Ashournia, Arezoo Akhlaghi.

**Project administration:** Masoumeh Ghasempour Alamdari.

**Resources:** Simin Khayatzadeh Kakhki, Ghazal Shariatpanahi.

**Software:** Niloufar Ghanbari.

**Validation:** Paniz Pourpashang, Malihe Sadat Rahimi Azghadi, Arezoo Akhlaghi.

**Writing – original draft:** Masoumeh Ghasempour Alamdari, Paniz Pourpashang, Simin Khayatzadeh Kakhki, Malihe Sadat Rahimi Azghadi, Parisa Ashournia.












