# Supplementary material for: Evaluating the efficacy and safety of macrolide therapy in primary ciliary dyskinesia: A systematic review
Source: Medicine (Baltimore). 2026 Aug 21;105(34):e50263. doi: 10.1097/MD.0000000000050263 (PMC13502839; doi:10.1097/MD.0000000000050263)
Supplement: Supplementary file 1 [file medi-105-e50263-s001.docx]

**Supplementary Table 1. PubMed search strategy**

| **Field** | **Search strategy / details** |
| --- | --- |
| Database | PubMed/MEDLINE |
| Search string | (("Primary Ciliary Dyskinesia"[Mesh] OR "primary ciliary dyskinesia"[tiab] OR PCD[tiab] OR "immotile cilia syndrome"[tiab] OR "Kartagener syndrome"[tiab]) AND ("Macrolides"[Mesh] OR macrolide*[tiab] OR azithromycin[tiab] OR clarithromycin[tiab] OR erythromycin[tiab])) |
| Limits | Publication date from January 1, 1990 to June 18, 2024; no language restriction. |
