# Supplementary material for: Evaluating the efficacy and safety of macrolide therapy in primary ciliary dyskinesia: A systematic review
Source: Medicine (Baltimore). 2026 Aug 21;105(34):e50263. doi: 10.1097/MD.0000000000050263 (PMC13502839; doi:10.1097/MD.0000000000050263)
Supplement: Supplementary file 2 [file medi-105-e50263-s002.docx]

**Supplementary Table 2. Scopus search strategy**

| **Field** | **Search strategy / details** |
| --- | --- |
| Database | Scopus |
| Search string | TITLE-ABS-KEY ( "primary ciliary dyskinesia" OR PCD OR "immotile cilia syndrome" OR "Kartagener syndrome" ) AND TITLE-ABS-KEY ( macrolide* OR azithromycin OR clarithromycin OR erythromycin ) |
| Limits | Publication year 1990 to 2024; no language restriction. |
