# Supplementary material for: Evaluating the efficacy and safety of macrolide therapy in primary ciliary dyskinesia: A systematic review
Source: Medicine (Baltimore). 2026 Aug 21;105(34):e50263. doi: 10.1097/MD.0000000000050263 (PMC13502839; doi:10.1097/MD.0000000000050263)
Supplement: Supplementary file 3 [file medi-105-e50263-s003.docx]

**Supplementary Table 3. Web of Science search strategy**

| **Field** | **Search strategy / details** |
| --- | --- |
| Database | Web of Science Core Collection |
| Search string | TS=("primary ciliary dyskinesia" OR PCD OR "immotile cilia syndrome" OR "Kartagener syndrome") AND TS=(macrolide* OR azithromycin OR clarithromycin OR erythromycin) |
| Limits | Timespan 1990 to June 18, 2024; all document types screened according to eligibility criteria. |
