# Supplementary material for: Evaluating the efficacy and safety of macrolide therapy in primary ciliary dyskinesia: A systematic review
Source: Medicine (Baltimore). 2026 Aug 21;105(34):e50263. doi: 10.1097/MD.0000000000050263 (PMC13502839; doi:10.1097/MD.0000000000050263)
Supplement: Supplementary file 4 [file medi-105-e50263-s004.docx]

**Supplementary Table 4. Embase search strategy**

| **Field** | **Search strategy / details** |
| --- | --- |
| Database | Embase |
| Search string | ('primary ciliary dyskinesia'/exp OR 'primary ciliary dyskinesia':ti,ab OR pcd:ti,ab OR 'immotile cilia syndrome':ti,ab OR 'kartagener syndrome':ti,ab) AND ('macrolide antibiotic agent'/exp OR macrolide*:ti,ab OR azithromycin:ti,ab OR clarithromycin:ti,ab OR erythromycin:ti,ab) |
| Limits | Publication year 1990 to 2024; no language restriction. |
