# Supplementary material for: Evaluating the efficacy and safety of macrolide therapy in primary ciliary dyskinesia: A systematic review
Source: Medicine (Baltimore). 2026 Aug 21;105(34):e50263. doi: 10.1097/MD.0000000000050263 (PMC13502839; doi:10.1097/MD.0000000000050263)
Supplement: Supplementary file 5 [file medi-105-e50263-s005.docx]

**Supplementary Table 5. Cochrane Library search strategy**

| **Field** | **Search strategy / details** |
| --- | --- |
| Database | Cochrane Library |
| Search string | ([mh "Primary Ciliary Dyskinesia"] OR "primary ciliary dyskinesia":ti,ab,kw OR PCD:ti,ab,kw OR "immotile cilia syndrome":ti,ab,kw OR "Kartagener syndrome":ti,ab,kw) AND ([mh Macrolides] OR macrolide*:ti,ab,kw OR azithromycin:ti,ab,kw OR clarithromycin:ti,ab,kw OR erythromycin:ti,ab,kw) |
| Limits | Publication date 1990 to June 18, 2024; trials and reviews screened according to eligibility criteria. |
