# Supplementary material for: Evaluating the efficacy and safety of macrolide therapy in primary ciliary dyskinesia: A systematic review
Source: Medicine (Baltimore). 2026 Aug 21;105(34):e50263. doi: 10.1097/MD.0000000000050263 (PMC13502839; doi:10.1097/MD.0000000000050263)
Supplement: Supplementary file 6 [file medi-105-e50263-s006.docx]

**Supplementary Table 6. Google Scholar and gray literature search strategy**

| **Field** | **Search strategy / details** |
| --- | --- |
| Sources | Google Scholar, ClinicalTrials.gov, WHO International Clinical Trials Registry Platform, allconferences.com, and OpenGrey |
| Google Scholar search terms | "primary ciliary dyskinesia" AND (macrolide OR azithromycin OR clarithromycin OR erythromycin); "Kartagener syndrome" AND (macrolide OR azithromycin OR clarithromycin OR erythromycin); "immotile cilia syndrome" AND (macrolide OR azithromycin OR clarithromycin OR erythromycin). |
| Trial registry / gray literature search terms | Primary ciliary dyskinesia; PCD; Kartagener syndrome; azithromycin; macrolide. |
| Screening approach | Records were screened against the same PICOS eligibility criteria used for the electronic database search. |
